# PhageLeads: Rapid Assessment of Phage Therapeutic Suitability Using an Ensemble Machine Learning Approach

**DOI:** 10.3390/v14020342

**Published:** 2022-02-08

**Authors:** Kumarasan Yukgehnaish, Heera Rajandas, Sivachandran Parimannan, Ravichandran Manickam, Kasi Marimuthu, Bent Petersen, Martha R. J. Clokie, Andrew Millard, Thomas Sicheritz-Pontén

**Affiliations:** 1 Center of Excellence for Omics-Driven Computational Biodiscovery (COMBio), AIMST University, Bedong 08100, Kedah, Malaysia; yukgehnaish91@gmail.com (K.Y.); sivachandran@aimst.edu.my (S.P.); ravichandran@aimst.edu.my (R.M.); kasi_marimuthu@aimst.edu.my (K.M.); bent.petersen@sund.ku.dk (B.P.); 2 GLOBE Institute, University of Copenhagen, 1165 Copenhagen, Denmark; 3 Department of Biotechnology, Faculty of Applied Sciences, AIMST University, Semeling 08100, Kedah, Malaysia; 4 Centre of Excellence for Vaccine Development (CoEVD), Faculty of Applied Sciences, AIMST University, Bedong 08100, Kedah, Malaysia; 5 Center for Evolutionary Hologenomics, University of Copenhagen, 1165 Copenhagen, Denmark; 6 Department Genetics and Genome Biology, University of Leicester, Leicester LE1 7RH, UK; mrjc1@leicester.ac.uk (M.R.J.C.); adm39@leicester.ac.uk (A.M.)

**Keywords:** phage therapy, AMR, lysogeny, machine learning, genomics

## Abstract

The characterization of therapeutic phage genomes plays a crucial role in the success rate of phage therapies. There are three checkpoints that need to be examined for the selection of phage candidates, namely, the presence of temperate markers, antimicrobial resistance (AMR) genes, and virulence genes. However, currently, no single-step tools are available for this purpose. Hence, we have developed a tool capable of checking all three conditions required for the selection of suitable therapeutic phage candidates. This tool consists of an ensemble of machine-learning-based predictors for determining the presence of temperate markers (integrase, Cro/CI repressor, immunity repressor, DNA partitioning protein A, and antirepressor) along with the integration of the ABRicate tool to determine the presence of antibiotic resistance genes and virulence genes. Using the biological features of the temperate markers, we were able to predict the presence of the temperate markers with high MCC scores (>0.70), corresponding to the lifestyle of the phages with an accuracy of 96.5%. Additionally, the screening of 183 lytic phage genomes revealed that six phages were found to contain AMR or virulence genes, showing that not all lytic phages are suitable to be used for therapy. The suite of predictors, PhageLeads, along with the integrated ABRicate tool, can be accessed online for in silico selection of suitable therapeutic phage candidates from single genome or metagenomic contigs.

## 1. Introduction

Phage therapy has been gaining ground in the field of medicine as a type of therapeutics for treating bacterial infections [1]. It is especially useful for treating infections from microbes that have acquired resistance to conventional antibiotics. These resistant microbes initially acquire resistance to antibiotics through genomic mutations caused by selective pressure in the environment (such as the presence of antibiotics), which can later be transferred to other bacteria through horizontal gene transfer [2], which allows these microbes to be able to counter the molecular action of the antibiotics [3]. Various clinical case reports have shown successful outcomes where phage therapy was used to treat a broad spectrum of bacterial infections such as prosthetic infections, musculoskeletal infections, urinary tract infections (UTI), septicemia, and biofilm infections [4,5,6,7,8,9]. The success of phage therapy is heavily dependent on the selection of suitable phage candidates, which ultimately comes down to the genome characterization of phages. However, this process is complicated due to the mosaicity of the phage genomes, leading to a lack of conserved regions and presence of many (>50%) hypothetical proteins with unknown functions in them [10,11,12]. These innate characteristics of phage genomes greatly reduce the effectiveness of conventional sequence alignment methods.

In order to mitigate any possible risks and adverse effects during phage therapy, a bioinformatic tool for the selection of suitable therapeutic phage candidates must be able to check for the following conditions in the phage genome [13]: (i) presence of temperate markers, (ii) presence of toxin and virulence genes, and (iii) presence of antimicrobial resistance genes. It is crucial that these markers and genes are absent in any phages intended for therapeutic use. However, no single-step tools that can be used for the selection of suitable therapeutic phage candidates are currently available. 

For condition (i), the determination of the presence of temperate markers in the phages ensures the exclusion of non-strictly lytic phages from being used in phage therapy. The mechanism of action of phage on a bacterial host depends on the life cycle of the phage, which can be classified into lytic, temperate, chronic, and pseudolysogenic cycles [14,15]. Out of these four lifestyles, lytic phages are considered as the most suitable candidates for phage therapy due to their rapid bactericidal effect. Besides that, there are greater risks associated with the usage of temperate phage in therapeutic treatment due to their ability to integrate “harmful” genes, such as antimicrobial resistance genes and virulence genes into the bacterial hosts through genome integration [16,17,18,19]. Besides that, temperate phages are able to provide superinfection immunity to the infected bacterial host, which prevents secondary infections by other phages, resulting in a decline in the replication capacity of the phage population [20]. This increases the risk of adverse effects compared to their intended therapeutic effect [1]. Currently, there are two widely used tools available for determining phage lifestyle, namely PHACTS and BACPHLIP. Both these tools employ similarity algorithms to annotate the proteomes of the phages and to classify their lifestyles based on their conserved protein domains using random forest classifier [21,22]. However, these methods still rely on sequence similarity algorithms such as HMMER3 [23] to determine the similarities between conserved protein domains of the phage of interest and other temperate phages in the database. This method is still bound to the limitations of efficiency of sequence similarity algorithms for phage genome characterization as described earlier. Instead, protein biological feature spaces such as di- and tri-peptide frequencies, isoelectric points, flexibility indices, and other generic protein features are found to be more effective in determining phage proteins’ functional similarities [24,25,26].

Although strictly lytic phages are preferred for therapeutic use, this does not mean that all lytic phages are excluded from the risk of adverse effects, as their genomes may still harbor antibiotic resistance genes and phage-encoded toxins and virulence genes [10,27], which requires checking for the conditions (ii) and (iii) described earlier. The primary method of detecting these genes is performed by comparing the ORF sequences of the candidates to the available databases. There are many comprehensive databases that are available to date for detecting these genes, such as the Comprehensive Antibiotic Resistance Database (CARD), Short, Better Representative Extract Dataset Antibiotic resistance (ShortBred AR), MEGARes, and National Database of Antibiotic Resistant Organism (NDARO) for the detection of antimicrobial genes [28,29,30,31]. Virulence factors are detected using databases such as ShortBred VF and Virulence Factor Database (VFDB) [29,32]. These databases can be accessed for determining the presence of antibiotic resistance genes and virulence genes individually. However, the ABRicate tool, which utilizes these multiple databases as a bundle, provides convenience for the researchers to detect these genes simultaneously [33].

Keeping in mind all the aforementioned conditions for selecting suitable therapeutic phage candidates and the limitations of existing tools, we have developed an online single-step tool for the determination of safe phage candidates using an ensemble of extreme gradient boosting technique-based predictors. These predictors utilize protein features to determine the presences of integrases, Cro/CI repressor proteins, immunity repressors, DNA partitioning protein A (ParA), and anti-repressor proteins in the phage candidates, along with the integration of the ABRicate tool to detect harmful genes, allowing the researchers to rapidly deselect phages to be used for phage therapy. 

## 2. Materials and Methods

### 2.1. Dataset Creation

Phage complete genomes dated prior to March 2018 were acquired from the NCBI RefSeq database in Genbank format. A total of 8368 phage GenBank files were obtained, out of which a total of 772,938 CDS protein sequences were extracted. From these sequences, any proteins with less than 30 amino acids were removed, along with hypothetical proteins and any proteins with unclear annotations, resulting in 188,080 protein sequences from 7686 phage genomes. The retained protein sequences were used for the training of the model. For the validation dataset, 63 phage genomes dated after March 2018 were obtained from the NCBI RefSeq database, with 45,231 protein sequences. For each predictor, the protein sequences were labeled based on their respective annotations. For example, for creating the integrase dataset, positive labels contained proteins that were annotated as “integrase”, “site-specific recombinase”, “tyrosine recombinase”, “serine recombinase” and other related keywords. Any proteins that did not belong to the positive dataset were labeled as negatives. Furthermore, proteins that were ambiguously annotated such as DNA binding protein, transcription regulator, repressors, and DNA partitioning proteins (with no subunits) were removed from the respective datasets. The keywords used for labeling the data and the resulting number of positive and negative labels are shown in Table 1. In order to ensure that the dataset is optimized for each of the predictors (not “one dataset fits all”), the datasets were created and filtered independent from each other. For example, for preparation of the integrase dataset, ambiguous annotations of the integrase proteins were removed from the dataset, while for the repressor protein dataset, ambiguous annotations of the repressor proteins were removed. 

**Figure 1:**
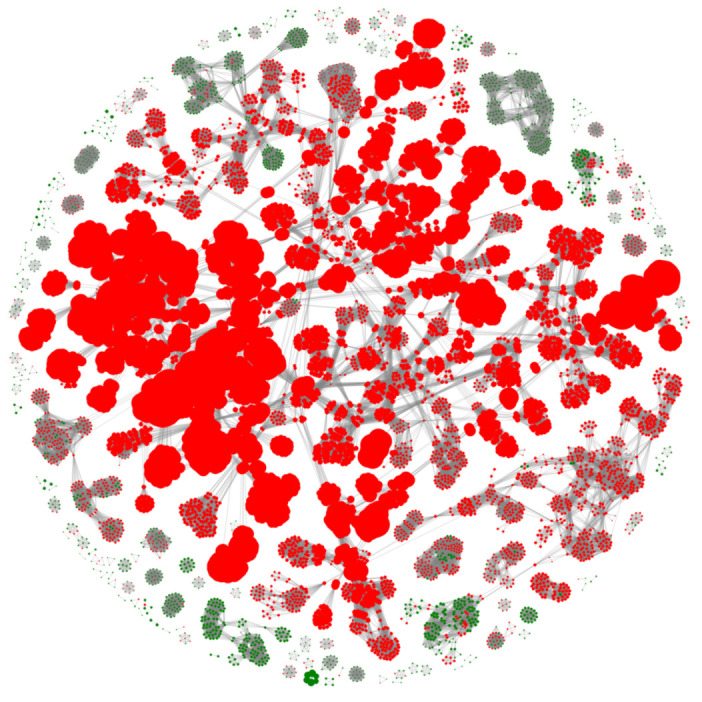
Network graph of the integrase dataset with green dots indicating positive labels and red dots indicating negative labels.

To prevent any misclassification of proteins to the positive and negative bins due to misannotation, a filtering step was included prior to training of the predictor model. To filter any possible mislabeled proteins, all the proteins in the positive and negative bins were aligned to themselves using DIAMOND [34] and clustered based on different bit score values (bitscore = 75, 100, 125). The results were then visualized as graphs using the python networkx module [35], with the positive and negative data as colored nodes and the respective bit scores as edges. An example network graph generated from the integrase dataset is shown in Figure 1. The network graph was used to visualize the protein clusters according to their respective labels, from which the misclassification, any negative label in positive clusters (<20%), and vice versa were removed from the dataset. If any of the misclassifications consisted of more than 20% of the total number of proteins in that cluster, all the proteins from that cluster were removed. This resulted in three filtered datasets, namely filtered_75, filtered_100, and filtered_125. The protein features of the original and three filtered datasets were subsequently extracted.

### 2.2. Feature Generation

A total of 1576 protein features were extracted from the proteins using custom scripts as described in [26]. The features included 400 dipeptide frequency, 100 reduced amino acid dipeptide frequency, 1000 reduced amino acid tripeptide frequency, 34 PROSITE domain features, and 42 generic protein features from the BioPython SeqUtils ProteinAnalysis module [36]. Selection of the final features used for each predictor was based on the importance of the features as calculated from the XGBoost module [37].

### 2.3. Training and Testing Using XGBoost

After the extraction of the protein features from the dataset, the dataset was split into training and testing sets with a ratio of 0.7 to 0.3. The protein features were passed to an extreme gradient boosting tool (XGBoost) to learn to differentiate between the positive and negative classes. XGBoost utilizes a gradient boosting framework to generate decision tree models, in which the new models are added sequentially to the ensemble based on the error values obtained from the previous models to decrease the total error value to the minimum. One common issue in machine learning models is the imbalance of classes in the training set; however, this was resolved by passing the scale positive weight parameter of the XGBoost module to compensate for the unbalanced classes. The scale positive weight for each dataset was calculated using the formula: scale positive weight = number of negative data/number of positive data. The training and testing of the model were performed in rounds. For each round, top features contributing to the correct prediction were selected by removing the 50 least contributing features, until the minimal number of features was reached. The final features were then selected based on the Matthews correlation coefficient (MCC) scores obtained from testing each of the models with the test dataset. The final predictors were trained using the optimal features and were validated using the validation dataset. 

### 2.4. 10-Fold Cross-Validation

In order to determine the learning ability of the model using the extracted features, 10-fold cross-validation was performed using two different methods. For the first method, a generic 10-fold cross-validation method was performed, using the StratifiedKFold module from the sklearn python module [38]. Instead of randomly grouping the datasets into 10 bins, in the second method, all the proteins were clustered using the cd-hit tool at low identity (60%), resulting in multiple clusters. The clusters were then grouped into 10 bins by making sure the proteins from the same clusters were always grouped into the same bins. This was performed to determine how well the predictor is able to predict across different clusters of proteins that were not included in the training. 

### 2.5. Screening for Virulence and Undesirable Genes

To screen the possible candidate phages for the presence of any undesirable genes such as virulence factors and antibiotic resistance genes, the ABRicate tool was used. ABRicate enabled the mass screening of predicted ORFs for detection of any undesirable genes by comparing the query sequences to different available databases, namely, NCBI, CARD, ARG-ANNOT, Resfinder, MEGARES, EcOH, PlasmidFinder, Ecoli_VF and VFDB [28,30,32,39,40,41,42,43]. This step was crucial in order to eliminate the possibility of lysogenic conversions and any undesirable recombination events that may occur between the candidate phages and the bacterial host, which in turn lead to the deleterious genes being assimilated into the bacterial host.

## 3. Results and Discussions

### 3.1. Selection of Best Dataset and Best Features

**Figure 2:**
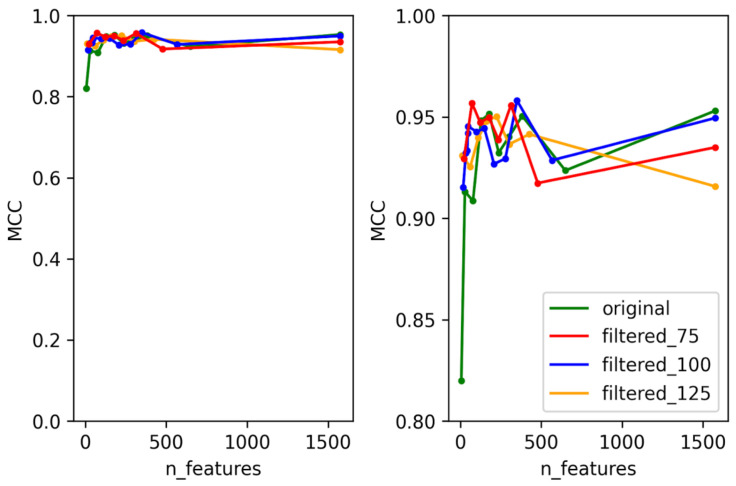
Test MCC of integrase predictor using original dataset and filtered datasets using different numbers of features. Graph on the right shows the magnified section of the original graph with MCC ranging from 0.8 to 1.

**Figure 3:**
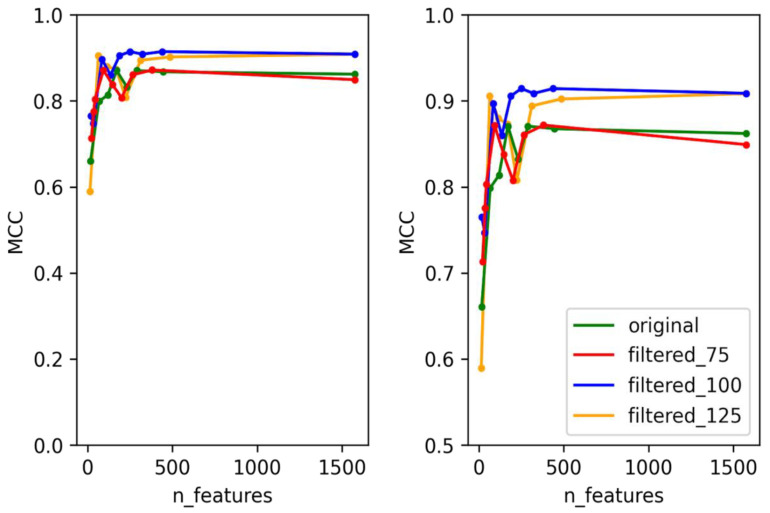
Test MCC of Cro/CI predictor using original dataset and filtered datasets using different numbers of features. Graph on the right shows the magnified section of the original graph with MCC ranging from 0.5 to 1.

**Figure 4:**
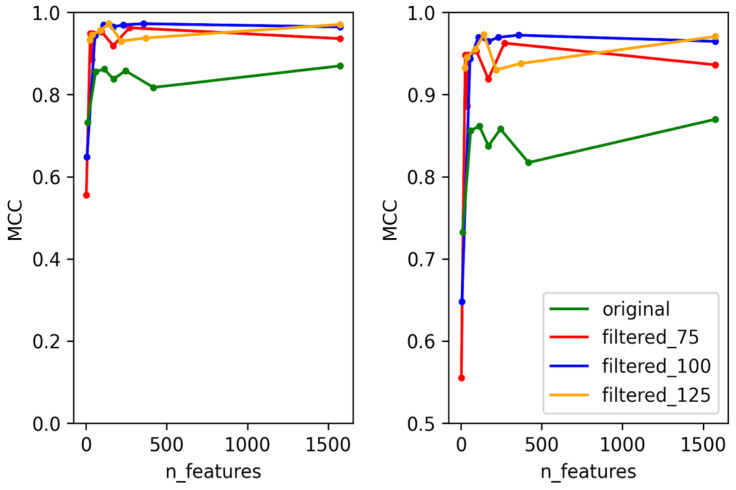
Test MCC of immunity repressor predictor using original dataset and filtered datasets using different numbers of features. Graph on the right shows the magnified section of the original graph with MCC ranging from 0.5 to 1.

**Figure 5:**
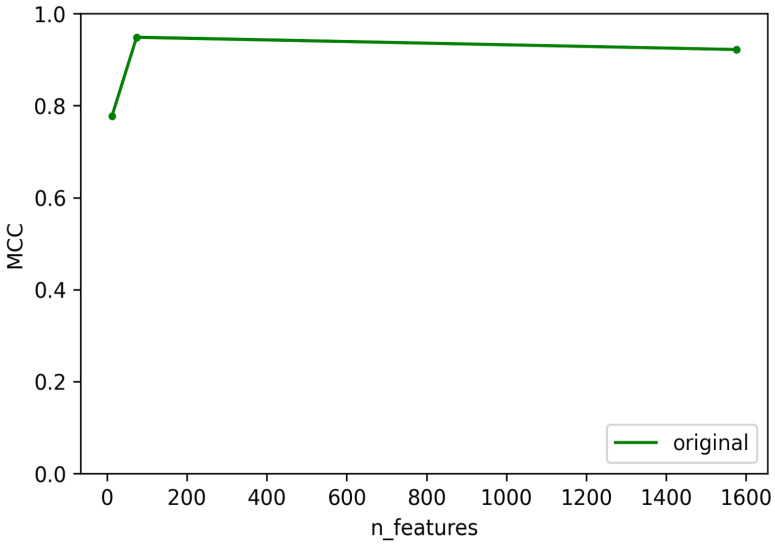
Test MCC of ParA predictor using original dataset and filtered datasets using different numbers of features.

**Figure 6:**
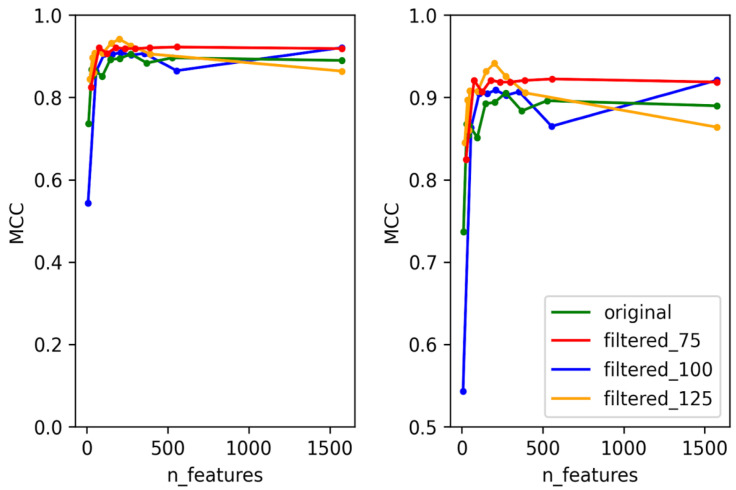
Test MCC of antirepressor predictor using original dataset and filtered datasets using different numbers of features. Graph on the right shows the magnified section of the original graph with MCC ranging from 0.5 to 1.

To create predictors with the best performance for each temperate marker (integrase, Cro/CI repressor, immunity repressor, parA, and antirepressor), two criteria were taken into consideration. First, for each temperate marker, we selected the dataset that can best represent the whole data used for training the model. This was performed by training and testing each of the models using four different datasets. These datasets consisted of the original dataset without any filtering and three datasets filtered based on bit scores (details provided in the methodology), except for ParA, which did not contain any annotation misclassification. The second consideration was to minimize the number of features used for each predictor and to select the best features that can be used to differentiate the positive and negative classes. In order to select the minimum number of features, the training and testing were performed in rounds. For each round, the number of features was decreased by a preset amount until the minimum number of features was reached. This method allowed us to select the best features while minimizing the probability of overfitting the model and to reduce the time taken for the predictor to predict. With these two considerations in mind, some level of compensation between the performance of the predictor and the number of features was allowed to select the optimal features that were used in the final predictor. The accuracy, F1 score, and area under the Curve (AUC) scores were calculated for each of the predictors, but the performance of each model was evaluated based on MCC scores, as these provide a much more reliable scoring metric for data with imbalanced classes [44]. The test MCC for each model for each round is shown in Figure 2, Figure 3, Figure 4, Figure 5 and Figure 6. For the integrase model, a mean MCC score of 0.92 was obtained using the original dataset, and 0.94 was obtained using the filtered_75, filtered_100, and filtered_125 datasets. For the Cro/CI repressor model, a mean MCC of 0.82 was obtained using the original and filtered_75 dataset, 0.87 using the filtered_100 dataset, and 0.85 using filtered_125 dataset. For the immunity repressor model, a mean MCC of 0.83, 0.88, 0.92, and 0.95 were obtained using original, filtered_75, filtered_100, and filtered_125, respectively. For the ParA model, mean MCC score of 0.88 was obtained using the original dataset. Finally, for antirepressor, mean MCC of 0.87, 0.91, 0.86, and 0.90 were obtained by using the original, filtered_75, filtered_100, and filtered_125 datasets, respectively. The performance metrics for each of the predictors are shown in Supplementary Table S1 (Performance metrics of temperate markers predictors). The MCCs obtained in each round were not linear to the number of features used for training, as the performance of the models fluctuated in each round when different numbers of features were used. It can be counterintuitive to observe the MCC to be lower in a model with the highest number of features compared to the model with the lower number of features. However, these fluctuations signify that not all the features play an equally significant role in the performance of the predictor and that the different combination of features used during learning explicitly affects the performance of the predictor. The final dataset and best features were then selected based on the aforementioned considerations. For the best dataset selection, the filtered_100 dataset was selected for integrase and Cro/CI repressor, the dataset filtered_125 was selected for immunity repressor and antirepressor, and the original dataset was selected for ParA. The number of selected features for the final predictors were 350, 251, 142, 74, and 201 for integrase, Cro/CI repressor, immunity repressor, ParA, and antirepressor, respectively.

### 3.2. Optimization and Validation of Final Predictor

The final predictors were trained using the selected datasets and features. To achieve the highest performances, the prediction thresholds were tuned to the value where the highest MCC scores were obtained using the training dataset. This was performed by iterating through the prediction threshold using incremental values and selecting the threshold with the highest MCC. With that, a prediction threshold of 0.252 for integrase predictor, 0.363 for Cro/CI repressor predictor, 0.472 for immunity repressor predictor, 0.03 for ParA predictor, and 0.389 for antirepressor predictor were obtained. Each predictor was then evaluated using the respective validation datasets. The validation MCC scores of each predictor were 0.92 for integrase (accuracy: 0.99, F1-score: 0.92), 0.71 for Cro/CI repressor (accuracy: 0.99, F1-score: 0.71), 0.87 for immunity repressor (accuracy: 0.99, F1-score: 0.86), 0.76 for ParA (accuracy: 0.99, F1-score: 0.75), and 0.92 for antirepressor (accuracy: 0.99, F1-score: 0.92). The optimized threshold values and the corresponding score metrics are shown in Table 2. 

### 3.3. 10-Fold Cross-Validation

**Figure 7:**
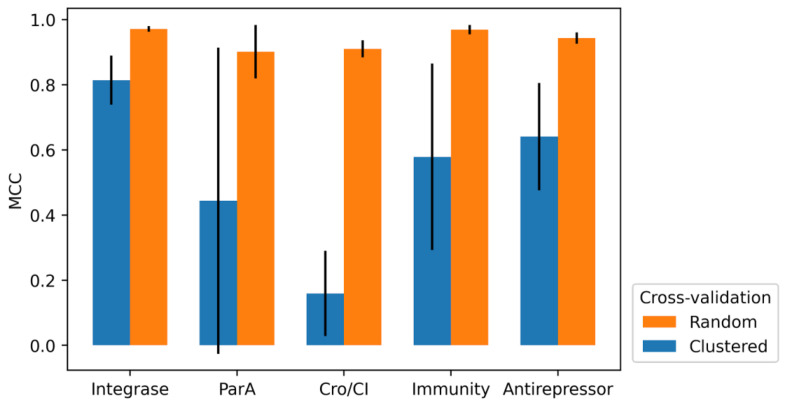
Mean MCC of random 10-fold cross-validation and clustered 10-fold cross-validation.

Cross-validation was performed to estimate the ability of the predictor to perform on unseen data. In this study, two types of 10-fold cross-validation were performed, namely, random 10-fold cross-validation and clustered 10-fold cross-validation. Random 10-fold cross-validation follows the generic 10-fold cross-validation steps where the datasets are randomly binned into 10 bins, and each bin is tested against the model trained using the remaining bins. The predictors were able to learn and predict across different bins successfully as indicated by the high MCC scores, 0.97 for integrase, 0.91 for Cro/CI repressor, 0.96 for immunity repressor, 0.90 for ParA, and 0.94 for antirepressor, as shown in Figure 7. The annotations of proteins are generally dependent on the sequence similarities and are heavily influenced by the variance in the sequences. In the case of phage proteins, the sequence similarities among a single type of protein can be as low as 40% [45]. To determine how these similarities affect the ability of the predictors to predict across different clusters of protein grouped according to their similarities, another round of 10-fold cross-validation was performed with a slight modification. Instead of randomly binning the dataset into 10 bins, the proteins were first clustered using cd-hit (similarities = 60%), and then the dataset were binned into 10 bins, making sure the proteins from the same clusters were always grouped into the same bin, with no cross-cluster proteins in any of the bins. The ability of the predictor to predict unseen clusters of proteins was relatively good for the integrase, with a mean MCC score of 0.81, followed by predictors with lower mean MCC scores of 0.64 for antirepressor, 0.58 for immunity repressor, 0.44 for ParA, and 0.16 for Cro/CI, as shown in Figure 7. The lower MCC scores obtained from the clustered 10-fold cross validation (ParA and Cro/CI repressor) may indicate lower conservation among the sequences of the respective protein markers, which ultimately reflects the importance of including a large dataset for protein predictions. If a particular cluster of protein from the positive data is not included during the training process, the ability of the predictor to predict that cluster of protein is greatly limited. 

### 3.4. Feature Importance

The protein features used in this study were found to be effective in differentiating various classes of proteins [26]. In total, 626 unique features were used for the final predictors, out of which 171 features were uniquely used for integrase, 90 features were uniquely used for Cro/CI repressor, 59 features for immunity repressor, 18 features for ParA, and 71 features for antirepressor. The detailed unique and shared features used for each of the predictors and their importance are shown in Supplementary Figure S1 (Feature importance scores of features used for temperate markers predictors). Reduced tripeptide frequencies were the most used features in all the predictors consisting of 50.32% of the total features, followed by dipeptide frequencies at 33.54%, reduced dipeptide frequencies at 9.58%, and generic protein features at 6.56%. Protein features derived from the sequences, such as amino acid compositions, aromaticity, flexibility, isoelectric points, and various other properties, were found to be effective in classification of protein and determination of their functions, especially in biological niches where the traditional annotation method using sequence similarities failed [46]. Various tools have been developed and used for effective classification of proteins using machine learning (ML) models by utilizing protein features as the initial input [47,48,49]. However, it is important to tailor the features used for each ML model according to the intended objective of the protein classifier, which was reflected from this current study where different protein classes require different combinations of features for the predictors to achieve optimal performance.

### 3.5. Prediction of Phage Lifestyle

To simulate the in silico selection of therapeutic phages based on the presence of temperate markers, we decided to use the phage lifestyle dataset to test the ability of the predictors to predict the presence of temperate markers, and to some extent, their lifestyle (temperate and lytic). The lifestyle prediction was performed with a non-syllogistic assumption that lytic phages will not contain any temperate markers. Although this is not entirely true, it provides an empirically validated dataset for comparing against available similar prediction tools. As the focus of PhageLeads is to assess the suitability of a virulent phage for clinical use, we have chosen a conservative approach where any phage that carries any of the chosen marker genes will be classified as temperate and not suitable. The phage lifestyle dataset was used for validation of BACPHLIP, a random forest-based phage lifestyle predictor that uses features obtained from the presence of 206 lysogeny markers using HMM protein sequence similarity calculations [22]. This dataset was originally compiled by Mavrich and Hatfull [50], consisting of 423 phages with an empirically validated lifestyle of the phages, with 240 temperate phages and 183 lytic phages. Performance of PhageLeads on the phage lifestyle dataset was good, with an MCC score of 0.92 (accuracy: 0.96, F1-score: 0.95). A total of 16 genomes were mispredicted, with seven false positives and nine false negatives. A confusion matrix of the prediction output is shown in Table 3.

Using the same test dataset, phage lifestyle predicted by BACPHLIP contained seven erroneous predictions. The higher accuracy of this predictor may be attributed to the lesser number of data used in the training and testing process of the tool compared to PhageLeads [50]. The training data from BACPHLIP only contained information from 634 phage genomes, encompassing five phage families. In contrast, 8368 phage genomes from 17 families were used to train PhageLeads, which provides a better representation of the currently existing phage sequence space. Phage lifestyle information obtained from literature reviews is not completely dependable, as the lifestyle of the phages may be generalized based on the type of phage or the reference genome used during the annotation. Other than that, the information on the numbers of experiments reproducibly performed to determine the phage lifestyle may not be readily available during review, which decreases the confidence of the actual lifestyle of the phages. When this lifestyle information is used during training of a model, the resulting predictions may not reflect the actual lifestyle of the phages. Hence, to prevent using such ambiguous information that contributes to biased predictions, no lifestyle information was included in the training of PhageLeads. Instead, the lifestyle of the phages was deduced purely from the existence of these lysogeny markers. From the phage lifestyle dataset used in this study, we observed that in some phages, although annotated as lytic phages, their genomes contained some of the temperate markers. As shown in Table 4, these genomes resulted in false positive predictions (lytic predicted as temperate) by PhageLeads. Although the presence of these predicted temperate markers in lytic phages do not always reflect the actual temperate lifestyle of the phages [51], the presence of these proteins in the phage genomes may still be a risk for the phages to be used in phage therapy.

### 3.6. Caveat in Phage Protein Predictors 

One of the major challenges during the construction of PhageLeads was the presence of ambiguously annotated proteins in the database. For example, some Cro/CI repressors were simply annotated as “transcriptional regulators” or “repressor”, some integrase proteins were annotated as “DNA-binding protein” or “recombinase”, while some ParA proteins/chromosome partitioning protein A were simply annotated as “Chromosome partitioning protein” or “DNA partitioning protein”. If these ambiguously annotated proteins were used for constructing ML models, the accuracy of the models would be severely affected. Hence, while constructing PhageLeads, some of these unresolved ambiguous proteins were removed from the training data. However, in doing so, some classes of proteins of interest might have been removed due to their consistent misannotation throughout the database, leading to misclassification of phage lifestyles, and subsequently leading to false predictions. To reduce the biases due to false and ambiguous annotations of proteins, we suggest exploration of three-dimensional structures of these proteins to resolve any misannotations, as the functions of proteins are highly correlated to their three-dimensional structures [52]. This idea will be ventured upon in the near future for further improving the performance of PhageLeads. 

### 3.7. AMR and Virulence Genes in Lytic Phages

Although purely lytic phages are preferred for phage therapy, the presence of virulence and antibiotic genes in the phage genome makes them unsuitable to be used for therapeutic applications. This is to avoid any unintended recombination events that might cause these genes to be transduced into the host genomes, increasing the risk of failure or even adversely affecting the outcome of the phage therapy. Additional screening processes using the ABRicate tool to detect the presence of undesirable genes have been incorporated in PhageLeads. To demonstrate the presence of such genes in the genome of lytic phages and that not all lytic phages are suitable for candidates for phage therapy, genomes of lytic phages from the phage lifestyle dataset were screened for antimicrobial resistance and virulence genes using ABRicate. Out of 183 lytic phages, five phages were found to encode for one or multiple virulence proteins such as increased serum survival proteins (ISS), Type III secretion system effectors, Cytolethal distending toxins, Shiga toxins, and NleG effectors. One of the phages was found to encode both antimicrobial resistance genes (chloramphenicol resistance) and virulence genes (ISS), as shown in Table 5. These lytic phages are not suitable to be used for phage therapy due to risk of adverse effects. 

## 4. Conclusions

By utilizing the protein features of these temperate markers, PhageLeads was able to predict the lifestyle of phages with high accuracy (96.2%). PhageLeads consists of five individual temperate protein predictors for the temperate markers, which predicts the presence of these markers in phage genomes. Based on the presence of either one or multiple markers, we were able to effectively classify the lifestyle of phages (lytic or temperate). Additionally, the lytic phage genomes were screened using ABRicate tool, in which some lytic phages were found to encode antimicrobial resistance and virulence proteins, deeming them unsafe for phage therapy. PhageLeads was able to predict the presence of temperate markers in a single phage in 1.6 s on average and was able to detect the resistance and virulence genes in an average of 8.1 s, compared to 2.3 s taken for BACPHLIP to predict the lifestyle of a single phage. PhageLeads is available as an online tool at www.phageleads.dk (Last accessed on 15 January 2022) as a part of the PhageCompass consortium (www.phagecompass.dk, Last accessed on 15 January 2022), making it easily accessible for researchers and as an effective tool for determining the suitability of phage for therapeutic use. Additionally, PhageLeads can also be used for predicting the presence of lysogenic markers for metagenomic contigs. 

## Figures and Tables

**Table 1:** Keywords used for labeling positive and negative data from annotated protein names and the resulting number of positive and negative labels in training and validation datasets.

Dataset	Keywords Used for Positive Labels	Number of Positive Labels	Number of Negative Labels
Integrase	Integrase, site-specific recombinase, tyrosine recombinase, serine recombinase, int, tyr recombinase, ser recombinase	Training: 2224 Validation: 485	Training: 156,132 Validation: 38,885
Cro/CI	Cro, CI, C1, CL	Training: 657 Validation: 132	Training: 156,170 Validation: 39,238
Immunity repressor	Immunity related repressor, immunity repressor, ImmR	Training: 754 Validation: 305	Training: 157,602 Validation: 39,065
ParA	DNA partitioning protein A, Chromosome partitioning protein A, ParA, partitioning protein A	Training: 78 Validation: 9	Training: 158,663 Validation: 39,435
Antirepressor	Antirepressor, anti-repressor, antirepressor Rha, Rha	Training: 945 Validation: 185	Training: 157,864 Validation: 39,275

**Table 2:** Optimized prediction threshold and validation score metrics of each predictor.

Predictors	Threshold	Validation MCC	Validation F1	Validation Accuracy
Integrase	0.252	0.92	0.92	0.99
Cro/CI	0.363	0.71	0.71	0.99
Immunity repressor	0.472	0.87	0.86	0.99
ParA	0.03	0.76	0.75	0.99
Antirepressor	0.389	0.92	0.92	0.99

**Table 3:** Confusion matrix of the prediction of phage lifestyle by using integrase, Cro/CI, immunity repressor, ParA, and antirepressor predictor.

	Lytic	Temperate
Lytic	177	7
Temperate	9	231

**Table 4:** Lytic phages that have been predicted as temperate phages (false positive) that contained actual and putative temperate markers.

Phage Genome	Protein Name	Protein ID	Predicted Marker	Remark
Salmonella phage SPN19 (NC_019417)	hypothetical protein	YP_006990261.1	Integrase	Blastp result shows high similarity (99.1%) to viral integrase family 4 [Salmonella Phage 37;YP_009221453.1]
Salmonella phage FSL SP-088 (NC_021780)	hypothetical protein	YP_008239914.1	Integrase	Blastp result shows high similarity (97.3%) to viral integrase family 4 [Salmonella Phage 37;YP_009221453.1]
Lactococcus phage 4268 (NC_004746)	DUF739 family protein	NP_839893.1	Cro/CI	DUF739/pfam05339 contains putative Cro/CI repressors
	helix-turn-helix transcriptional regulator	NP_839899.1	Cro/CI	HTH-transcriptional regulator/repressor/HTH -containing proteins are common ambiguous annotations of Cro/CI
	putative antirepressor	NP_839894	Antirepressor	Lytic phage with lysogeny marker
Lactobacillus phage Lc-Nu (NC_007501)	CI-like repressor	YP_358780.1	Cro/CI	Lytic phage with lysogeny marker
	Cro-like repressor	YP_358781.1	Cro/CI	Lytic phage with lysogeny marker
Mycobacterium phage LRRHood (GQ303262)	immunity repressor	ACU41572.1	Immunity repressor	Lytic phage with lysogeny marker
Mycobacterium phage Alice (JF704092)	hypothetical protein	AEJ94305	Immunity repressor	Blastp result shows high similarity(98.82%) to immunity repressor [Mycobacterium phage Phox; ATN91327.1]

**Table 5:** Antimicrobial resistance and virulence genes obtained by screening the genome of lytic phages using ABRicate.

Phage Genome	Gene	Product	Resistance/Virulence	Database Source
Stx2 converting phage vB_EcoP_24B (NC_027984)	CatA1	type A-1 chloramphenicol O-acetyltransferase	Chloramphenicol resistance	NCBI, CARD, ARGANNOT, resfinder, megares
	iss2	Increase serum survival protein	Virulence	ecoli_vf
Enterobacteria phage lambda (NC_001416)	iss2	Increase serum survival protein	Virulence	ecoli_vf
Phage cdtI (NC_009514)	nleH1	Type III secretion system effector NleH1	Virulence	ecoli_vf
	cif	Type III secretion system effector Cif cyclomodulin	Virulence	ecoli_vf
	cdtA	Cytolethal distending toxin subunit A	Virulence	ecoli_vf
	cdtB	Cytolethal distending toxin subunit B	Virulence	ecoli_vf
	cdtC	Cytolethal distending toxin subunit C	Virulence	ecoli_vf
Enterobacteria phage YYZ-2008 (NC_011356)	stx1A	Shiga toxin 1 subunit A	Virulence	ecoli_vf
	stx1B	Shiga-like toxin 1 subunit B	Virulence	ecoli_vf
	nleG6-3	NleG Type 3 Effectors	Virulence	ecoli_vf
	nleG5-1	NleG Type 1 Effectors	Virulence	ecoli_vf
Escherichia phage TL-2011c (NC_019442)	stx2A	Shiga toxin 2 subunit A	Virulence	ecoli_vf
	stx2B	Shiga-like toxin II subunit B	Virulence	ecoli_vf
	iss2	Increase serum survival protein	Virulence	ecoli_vf
Enterobacteria phage HK629 (NC_019711)	iss2	Increase serum survival protein	Virulence	ecoli_vf

## Data Availability

All the data used in this study are available at: https://sid.erda.dk/sharelink/bW2RmLjL6A accessed on 20 January 2022.

## Supplementary Materials

The following supporting information can be downloaded at: https://www.mdpi.com/article/10.3390/v14020342/s1, Figure S1: Feature importance scores of features used for temperate markers predictors; Table S1: Performance metrics of temperate markers predictors.

## References

[1] Gordillo Altamirano F.L., Barr J.J. (2019). Phage Therapy in the Postantibiotic Era. Clin. Microbiol. Rev..

[2] Sundin G.W., Bender C.L. (1996). Dissemination of the StrA-StrB Streptomycin-Resistance Genes among Commensal and Pathogenic Bacteria from Humans, Animals, and Plants. Mol. Ecol..

[3] Nuti R., Goud N.S., Saraswati A.P., Alvala R., Alvala M. (2017). Antimicrobial Peptides: A Promising Therapeutic Strategy in Tackling Antimicrobial Resistance. Curr. Med. Chem..

[4] Ferry T., Kolenda C., Batailler C., Gustave C.-A., Lustig S., Malatray M., Fevre C., Josse J., Petitjean C., Chidiac C. (2020). Phage Therapy as Adjuvant to Conservative Surgery and Antibiotics to Salvage Patients with Relapsing, S. Aureus Prosthetic Knee Infection. Front. Med..

[5] Jault P., Leclerc T., Jennes S., Pirnay J.P., Que Y.-A., Resch G., Rousseau A.F., Ravat F., Carsin H., Le Floch R. (2019). Efficacy and Tolerability of a Cocktail of Bacteriophages to Treat Burn Wounds Infected by Pseudomonas Aeruginosa (PhagoBurn): A Randomised, Controlled, Double-Blind Phase 1/2 Trial. Lancet Infect. Dis..

[6] Aslam S., Lampley E., Wooten D., Karris M., Benson C., Strathdee S., Schooley R.T. (2020). Lessons Learned From the First 10 Consecutive Cases of Intravenous Bacteriophage Therapy to Treat Multidrug-Resistant Bacterial Infections at a Single Center in the United States. Open Forum Infect. Dis..

[7] Leitner L., Ujmajuridze A., Chanishvili N., Goderdzishvili M., Chkonia I., Rigvava S., Chkhotua A., Changashvili G., McCallin S., Schneider M.P. (2021). Intravesical Bacteriophages for Treating Urinary Tract Infections in Patients Undergoing Transurethral Resection of the Prostate: A Randomised, Placebo-Controlled, Double-Blind Clinical Trial. Lancet Infect. Dis..

[8] Onsea J., Soentjens P., Djebara S., Merabishvili M., Depypere M., Spriet I., De Munter P., Debaveye Y., Nijs S., Vanderschot P. (2019). Bacteriophage Application for Difficult-to-Treat Musculoskeletal Infections: Development of a Standardized Multidisciplinary Treatment Protocol. Viruses.

[9] Doub J.B. (2020). Bacteriophage Therapy for Clinical Biofilm Infections: Parameters That Influence Treatment Protocols and Current Treatment Approaches. Antibiotics.

[10] Philipson C.W., Voegtly L.J., Lueder M.R., Long K.A., Rice G.K., Frey K.G., Biswas B., Cer R.Z., Hamilton T., Bishop-Lilly K.A. (2018). Characterizing Phage Genomes for Therapeutic Applications. Viruses.

[11] Wan X., Hendrix H., Skurnik M., Lavigne R. (2021). Phage-Based Target Discovery and Its Exploitation towards Novel Antibacterial Molecules. Curr. Opin. Biotechnol..

[12] Lima-Mendez G., Van Helden J., Toussaint A., Leplae R. (2008). Reticulate Representation of Evolutionary and Functional Relationships between Phage Genomes. Mol. Biol. Evol..

[13] Doub J.B. (2021). Risk of Bacteriophage Therapeutics to Transfer Genetic Material and Contain Contaminants Beyond Endotoxins with Clinically Relevant Mitigation Strategies. Infect. Drug Resist..

[14] Clokie M.R.J., Millard A.D., Letarov A.V., Heaphy S. (2011). Phages in Nature. Bacteriophage.

[15] Mäntynen S., Laanto E., Oksanen H.M., Poranen M.M., Díaz-Muñoz S.L. (2021). Black Box of Phage–Bacterium Interactions: Exploring Alternative Phage Infection Strategies. Open Biol..

[16] Colavecchio A., Cadieux B., Lo A., Goodridge L.D. (2017). Bacteriophages Contribute to the Spread of Antibiotic Resistance Genes among Foodborne Pathogens of the Enterobacteriaceae Family—A Review. Front. Microbiol..

[17] Dąbrowska K., Abedon S.T. (2019). Pharmacologically Aware Phage Therapy: Pharmacodynamic and Pharmacokinetic Obstacles to Phage Antibacterial Action in Animal and Human Bodies. Microbiol. Mol. Biol. Rev..

[18] Moon K., Jeon J.H., Kang I., Park K.S., Lee K., Cha C.-J., Lee S.H., Cho J.-C. (2020). Freshwater Viral Metagenome Reveals Novel and Functional Phage-Borne Antibiotic Resistance Genes. Microbiome.

[19] Partridge S.R., Kwong S.M., Firth N., Jensen S.O. (2018). Mobile Genetic Elements Associated with Antimicrobial Resistance. Clin. Microbiol. Rev..

[20] Hassan A.Y., Lin J.T., Ricker N., Anany H. (2021). The Age of Phage: Friend or Foe in the New Dawn of Therapeutic and Biocontrol Applications?. Pharmaceuticals.

[21] McNair K., Bailey B.A., Edwards R.A. (2012). PHACTS, a Computational Approach to Classifying the Lifestyle of Phages. Bioinformatics.

[22] Hockenberry A.J., Wilke C.O. (2021). BACPHLIP: Predicting Bacteriophage Lifestyle from Conserved Protein Domains. PeerJ.

[23] Mistry J., Finn R.D., Eddy S.R., Bateman A., Punta M. (2013). Challenges in Homology Search: HMMER3 and Convergent Evolution of Coiled-Coil Regions. Nucleic Acids Res..

[24] Charoenkwan P., Kanthawong S., Schaduangrat N., Yana J., Shoombuatong W. (2020). PVPred-SCM: Improved Prediction and Analysis of Phage Virion Proteins Using a Scoring Card Method. Cells.

[25] Cantu V.A., Salamon P., Seguritan V., Redfield J., Salamon D., Edwards R.A., Segall A.M. (2020). PhANNs, a Fast and Accurate Tool and Web Server to Classify Phage Structural Proteins. PLOS Comput. Biol..

[26] Sirén K., Millard A., Petersen B., Gilbert M.T.P., Clokie M.R.J., Sicheritz-Pontén T. (2021). Rapid Discovery of Novel Prophages Using Biological Feature Engineering and Machine Learning. NAR Genom. Bioinforma..

[27] Hyman P. (2019). Phages for Phage Therapy: Isolation, Characterization, and Host Range Breadth. Pharmaceuticals.

[28] Jia B., Raphenya A.R., Alcock B., Waglechner N., Guo P., Tsang K.K., Lago B.A., Dave B.M., Pereira S., Sharma A.N. (2017). CARD 2017: Expansion and Model-Centric Curation of the Comprehensive Antibiotic Resistance Database. Nucleic Acids Res..

[29] Kaminski J., Gibson M.K., Franzosa E.A., Segata N., Dantas G., Huttenhower C. (2015). High-Specificity Targeted Functional Profiling in Microbial Communities with ShortBRED. PLOS Comput. Biol..

[30] Doster E., Lakin S.M., Dean C.J., Wolfe C., Young J.G., Boucher C., Belk K.E., Noyes N.R., Morley P.S. (2020). MEGARes 2.0: A Database for Classification of Antimicrobial Drug, Biocide and Metal Resistance Determinants in Metagenomic Sequence Data. Nucleic Acids Res..

[31] National Database of Antibiotic Resistant Organisms (NDARO)—Pathogen Detection—NCBI. https://www.ncbi.nlm.nih.gov/pathogens/antimicrobial-resistance/.

[32] Chen L., Zheng D., Liu B., Yang J., Jin Q. (2016). VFDB 2016: Hierarchical and Refined Dataset for Big Data Analysis--10 Years On. Nucleic Acids Res..

[33] Seemann T. (2021). ABRicate.

[34] Buchfink B., Reuter K., Drost H.-G. (2021). Sensitive Protein Alignments at Tree-of-Life Scale Using DIAMOND. Nat. Methods.

[35] Hagberg A., Swart P., S Chult D. Exploring Network Structure, Dynamics, and Function Using Networkx. Proceedings of the 7th Python in Science Conference.

[36] Cock P.J.A., Antao T., Chang J.T., Chapman B.A., Cox C.J., Dalke A., Friedberg I., Hamelryck T., Kauff F., Wilczynski B. (2009). Biopython: Freely Available Python Tools for Computational Molecular Biology and Bioinformatics. Bioinformatics.

[37] Chen T., Guestrin C. (2016). XGBoost: A Scalable Tree Boosting System. Proceedings of the 22nd ACM SIGKDD International Conference on Knowledge Discovery and Data Mining.

[38] Pedregosa F., Varoquaux G., Gramfort A., Michel V., Thirion B., Grisel O., Blondel M., Prettenhofer P., Weiss R., Dubourg V. (2011). Scikit-Learn: Machine Learning in Python. J. Mach. Learn. Res..

[39] Carattoli A., Zankari E., García-Fernández A., Voldby Larsen M., Lund O., Villa L., Møller Aarestrup F., Hasman H. (2014). In Silico Detection and Typing of Plasmids Using PlasmidFinder and Plasmid Multilocus Sequence Typing. Antimicrob. Agents Chemother..

[40] Feldgarden M., Brover V., Haft D.H., Prasad A.B., Slotta D.J., Tolstoy I., Tyson G.H., Zhao S., Hsu C.-H., McDermott P.F. (2019). Validating the AMRFinder Tool and Resistance Gene Database by Using Antimicrobial Resistance Genotype-Phenotype Correlations in a Collection of Isolates. Antimicrob. Agents Chemother..

[41] Gupta S.K., Padmanabhan B.R., Diene S.M., Lopez-Rojas R., Kempf M., Landraud L., Rolain J.-M. (2014). ARG-ANNOT, a New Bioinformatic Tool to Discover Antibiotic Resistance Genes in Bacterial Genomes. Antimicrob. Agents Chemother..

[42] Ingle D.J., Valcanis M., Kuzevski A., Tauschek M., Inouye M., Stinear T., Levine M.M., Robins-Browne R.M., Holt K.E. (2016). In Silico Serotyping of E. Coli from Short Read Data Identifies Limited Novel O-Loci but Extensive Diversity of O:H Serotype Combinations within and between Pathogenic Lineages. Microb. Genom..

[43] Zankari E., Hasman H., Cosentino S., Vestergaard M., Rasmussen S., Lund O., Aarestrup F.M., Larsen M.V. (2012). Identification of Acquired Antimicrobial Resistance Genes. J. Antimicrob. Chemother..

[44] Chicco D., Jurman G. (2020). The Advantages of the Matthews Correlation Coefficient (MCC) over F1 Score and Accuracy in Binary Classification Evaluation. BMC Genom..

[45] Dion M.B., Oechslin F., Moineau S. (2020). Phage Diversity, Genomics and Phylogeny. Nat. Rev. Microbiol..

[46] Ofer D., Linial M. (2015). ProFET: Feature Engineering Captures High-Level Protein Functions. Bioinformatics.

[47] Cao D.-S., Xu Q.-S., Liang Y.-Z. (2013). Propy: A Tool to Generate Various Modes of Chou’s PseAAC. Bioinformatics.

[48] Nanni L., Lumini A., Brahnam S. (2014). An Empirical Study of Different Approaches for Protein Classification. Sci. World J..

[49] van den Berg B.A., Reinders M.J., Roubos J.A., Ridder D. (2014). de SPiCE: A Web-Based Tool for Sequence-Based Protein Classification and Exploration. BMC Bioinform..

[50] Mavrich T.N., Hatfull G.F. (2017). Bacteriophage Evolution Differs by Host, Lifestyle and Genome. Nat. Microbiol..

[51] Shitrit D., Hackl T., Laurenceau R., Raho N., Carlson M.C.G., Sabehi G., Schwartz D.A., Chisholm S.W., Lindell D. (2021). Genetic Engineering of Marine Cyanophages Reveals Integration but Not Lysogeny in T7-like Cyanophages. ISME J..

[52] Jumper J., Evans R., Pritzel A., Green T., Figurnov M., Ronneberger O., Tunyasuvunakool K., Bates R., Žídek A., Potapenko A. (2021). Highly Accurate Protein Structure Prediction with AlphaFold. Nature.

