# Supplementary material for: PhageLeads: Rapid Assessment of Phage Therapeutic Suitability Using an Ensemble Machine Learning Approach
Source: Viruses. 2022 Feb 8;14(2):342. doi: 10.3390/v14020342 (PMC8879740; doi:10.3390/v14020342)
Supplement: Supplementary file 1 [file viruses-14-00342-s001.zip › viruses-1578960-SI/viruses-1578960 Supplementary_data.pdf]

Table S1 : Performance metrics of temperate markers predictors

Predictor	*Dataset*	*Mean MCC*	*Mean F1*	*Mean AUC*	*Mean Accuracy*
Integrase	Original	0.92	0.92	0.94	0.99
	Filtered_75	0.94	0.94	0.95	0.99
	Filtered_100	0.94	0.94	0.95	0.99
	Filtered_125	0.94	0.94	0.95	0.99
Cro/CI	Original	0.82	0.81	0.86	0.99
	Filtered_75	0.82	0.81	0.85	0.99
	Filtered_100	0.87	0.86	0.9	0.99
	Filtered_125	0.85	0.83	0.88	0.99
Immunity repressor	Original	0.83	0.83	0.9	0.99
	Filtered_75	0.88	0.88	0.91	0.99
	Filtered_100	0.92	0.91	0.93	0.99
	Filtered_125	0.95	0.94	0.96	0.99
ParA	Original	0.88	0.88	0.9	0.99
Antirepressor	Original	0.87	0.86	0.9	0.99
	Filtered_75	0.91	0.9	0.92	0.99
	Filtered_100	0.86	0.85	0.89	0.99
	Filtered_125	0.9	0.89	0.91	0.99

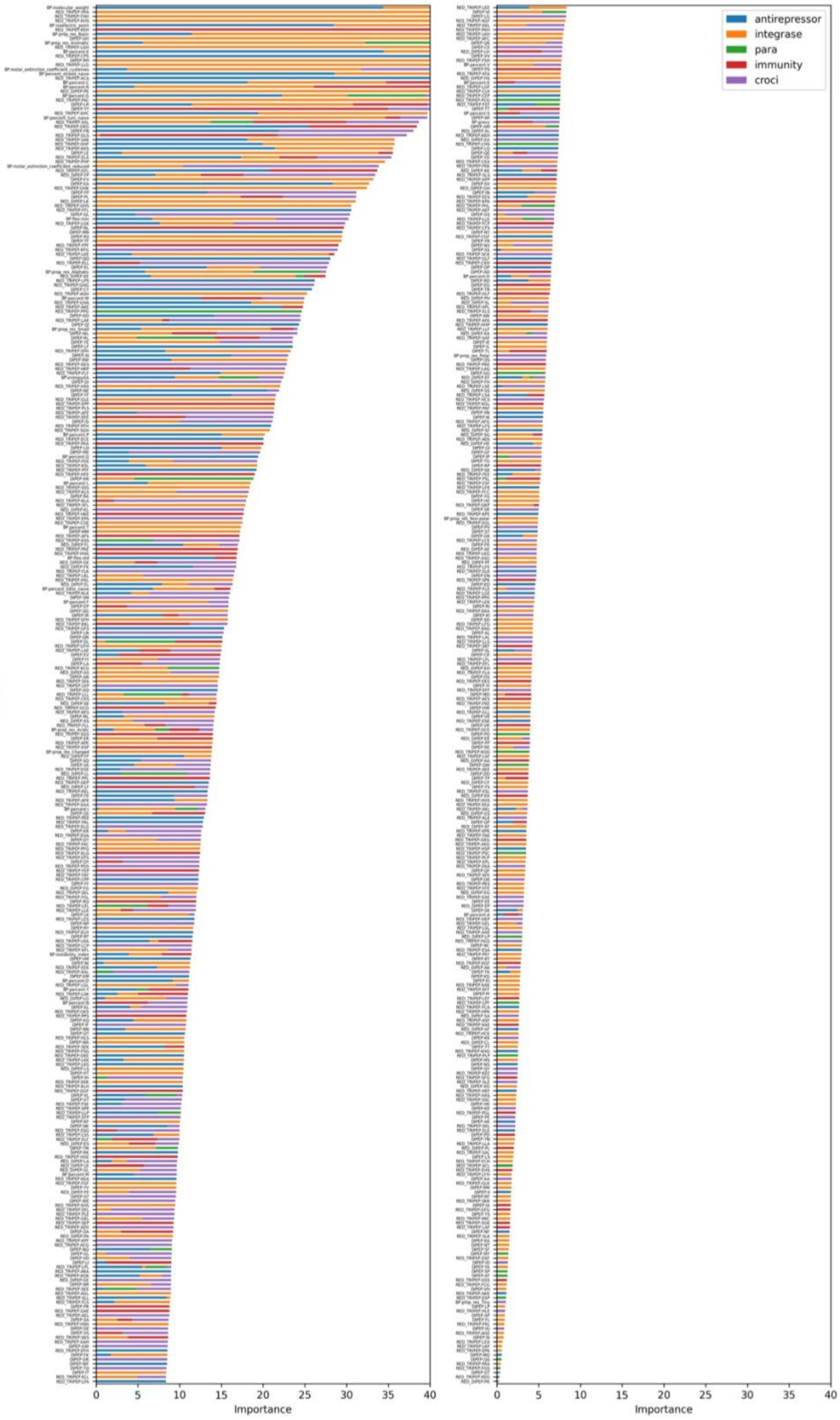

Figure S1: Feature importance scores of features used for temperate markers predictors
